# ATAV: a comprehensive platform for population-scale genomic analyses

**DOI:** 10.1186/s12859-021-04071-1

**Published:** 2021-03-23

**Authors:** Zhong Ren, Gundula Povysil, Joseph A. Hostyk, Hongzhu Cui, Nitin Bhardwaj, David B. Goldstein

**Affiliations:** Institute for Genomic Medicine, Columbia University Irving Medical Center, New York, NY 10032 USA

**Keywords:** Association testing, Genome analysis, Gene discovery, Diagnostic, Web platform

## Abstract

**Background:**

A common approach for sequencing studies is to do joint-calling and store variants of all samples in a single file. If new samples are continually added or controls are re-used for several studies, the cost and time required to perform joint-calling for each analysis can become prohibitive.

**Results:**

We present ATAV, an analysis platform for large-scale whole-exome and whole-genome sequencing projects. ATAV stores variant and per site coverage data for all samples in a centralized database, which is efficiently queried by ATAV to support diagnostic analyses for trios and singletons, as well as rare-variant collapsing analyses for finding disease associations in complex diseases. Runtime logs ensure full reproducibility and the modularized ATAV framework makes it extensible to continuous development. Besides helping with the identification of disease-causing variants for a range of diseases, ATAV has also enabled the discovery of disease-genes by rare-variant collapsing on datasets containing more than 20,000 samples. Analyses to date have been performed on data of more than 110,000 individuals demonstrating the scalability of the framework.

To allow users to easily access variant-level data directly from the database, we provide a web-based interface, the ATAV data browser (http://atavdb.org/). Through this browser, summary-level data for more than 40,000 samples can be queried by the general public representing a mix of cases and controls of diverse ancestries. Users have access to phenotype categories of variant carriers, as well as predicted ancestry, gender, and quality metrics. In contrast to many other platforms, the data browser is able to show data of newly-added samples in real-time and therefore evolves rapidly as more and more samples are sequenced.

**Conclusions:**

Through ATAV, users have public access to one of the largest variant databases for patients sequenced at a tertiary care center and can look up any genes or variants of interest. Additionally, since the entire code is freely available on GitHub, ATAV can easily be deployed by other groups that wish to build their own platform, database, and user interface.

## Background

Diagnostic and cohort sequencing studies benefit from the analysis of a large number of samples combined with similarly processed controls. A common approach to reach the necessary scale for analysis is to use a joint-calling procedure and store all samples in a single VCF file [1, 2]. While effective in allowing a single analysis of all samples included in the single VCF file, this approach has significant limitations. Perhaps most importantly, this approach is not amenable to ongoing analyses as new samples become available. Moreover, when projects combine multiple cohorts that were not sequenced together and in which controls might be re-used for several studies, the cost and time required to perform joint-calling for each analysis can become prohibitive. In addition to these considerations, typical sequencing file formats (VCF, BAM) place a sizeable overhead in moving these data from physical storage to the compute nodes for dynamic and multi user analysis needs. Furthermore, standard diagnostic and case–control studies leverage a range of filtering parameters, including variant calling (genotype quality, read coverage), variant annotation (gene, effect), internal population frequencies (minor allele frequency, genotype frequency) and external dataset filters (gnomAD [3], RVIS [4]) to identify "qualifying variants" that meet a specific set of user-defined criteria. These sophisticated needs require systematic logging and version control for re-analyses and reproducibility. As the data size and number of simultaneous users increase, ad-hoc analyses become prohibitively inefficient in the conventional single joint-genotyped VCF framework.

**Fig. 1:**
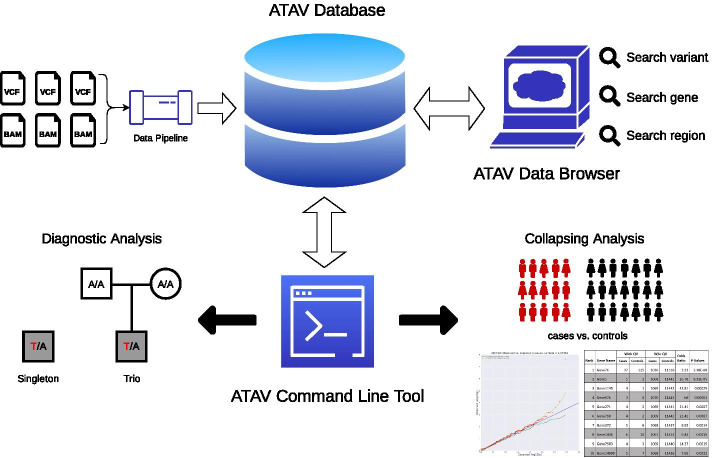
ATAV platform framework overview: data extracted from single-sample VCF and BAM files is stored in the ATAV database. The ATAV data browser and the ATAV command line tool retrieve information from the ATAV database for variant look-up, diagnostic analyses, and association studies using rare-variant collapsing

To address these constraints and dynamic analyses needs, we have developed ATAV (Analysis Tool for Annotated Variants, see Fig. 1) to streamline genomic analysis needs ranging from the standard diagnostic case interpretation to large-scale cohort analyses for disease-associated gene discovery. The ATAV platform is built on an open-source relational database. The database (ATAVDB) is configured with a feature allowing data replication across a cluster of nodes. ATAVDB contains sample level variant data, read coverage data, variant annotation data, external annotation data, and metadata. A data pipeline toolkit provided with the code extracts variants, annotations and associated quality data from VCF files and the coverage and genotype quality from BAM files. The Institute for Genomic Medicine (IGM) at Columbia University currently has data for more than 100 K whole exomes, and the coding-regions for over 10 K whole genomes stored in ATAVDB. It contains over 24 billion variant calls from over 220 million distinct genomic co-ordinates and read coverage information for all samples.

Several other tools and systems have been developed that address some of the same needs. For example, GORpipe [5] offers a fast way to query variant data, but does not offer easy pipelines for performing diagnostic variant prioritization or association studies. TileDB-VCF (https://github.com/TileDB-Inc/TileDB-VCF) offers a solution for simple processing, storage, and querying of data derived from single sample VCF files, but additional analyses have to be implemented by the user. Other frameworks such as Glow (https://github.com/projectglow/glow) and Hail (Hail Team. Hail 0.2. https://github.com/hail-is/hail) recommend jointly-called variant files as input, but offer functions for more complex analyses such as association tests. ATAV is, to the best of our knowledge, the only one that takes single sample files as input and still offers tools that can easily be used to perform full diagnostic analyses and rare-variant association tests. Furthermore, ATAV offers a web-interface and API that offers users access to a rich catalogue of variants detected in patients with a variety of diseases.

## Implementation

### Database

**Fig. 2:**
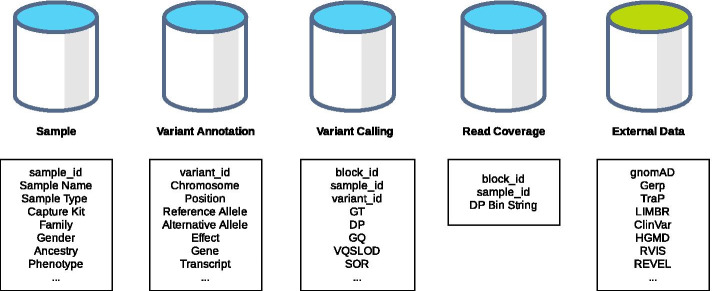
Schema for the ATAV core database (blue) and external databases (green)

We use Percona Server for MySQL and its high-performance storage engine Percona TokuDB to improve scalability and operational efficiency. In the database, we store a universal variant list across all samples, annotation data that is annotated through ClinEff [6], sample-level variant calls and associated quality metrics, as well as all sites’ coverage data for inferring reference alleles at non-call sites. In addition, ATAV has standardized code for incorporating any external data that is gene-based, site-based, or variant-based. ATAVDB currently stores external databases such as allele frequencies from gnomAD [3], ExAC [7], or, DiscovEHR [8]; scores such as TraP [9], LIMBR [10], MTR [11], RVIS [4], subRVIS [12], REVEL [13], PrimateAI [14], CCR [15]; and clinical annotations from ClinVar [16, 17], ClinGen [18], HGMD [19], and OMIM (see Fig. 2).

Variants, annotations and associated quality data are extracted from single-sample VCF files. A custom script checks whether a different representation of the same variant already exists in the database before adding a new variant to the database to ensure the same variant is represented identically in all samples.

**Fig. 3:**
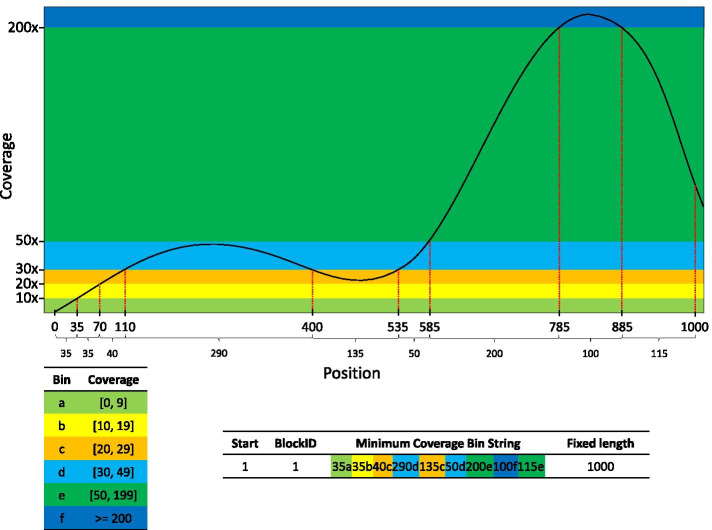
Efficient storage of coverage information: the per site coverage value is converted into a fixed 1000 base pair length bin string by first converting coverage values into bins (**a**–**f**) and then using a run-length encoding procedure to further compress data within fixed 1000 bp block regions by summarizing consecutive coverage values within the same bin

For efficiently storing coverage information for every site and every sample, the ATAV data pipeline parses the BAM files to generate read coverage data and converts site coverage values into binned values: a [0–9]; b [10–19]; c [20–29]; d [30–49]; e [50–199]; f ≥ 200. A run-length encoding procedure is used to further compress data within fixed 1000 bp block regions (see Fig. 3). This method reduces the data size by about 1000 times making it possible to store the coverage information for more than 100 K samples. Years of applied use have helped us to identify the information that is most often required for the standard genetic analyses performed as part of both diagnostic genetic studies and gene discovery. For example, in diagnostic analyses for identifying de novo mutations in affected children, it is necessary to know that the parental samples have sufficient coverage at the relevant site, but not necessary to know the precise number of reads, leading to the binning strategy for coverage described above. For the vast majority of applications, we have found that the necessary information can be economically stored and retrieved as described.

### Platform architecture

**Fig. 4:**
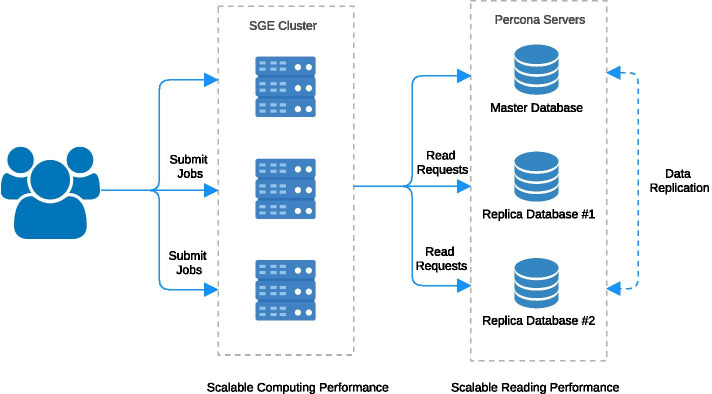
Platform architecture: the user submits jobs to an SGE cluster. The individual ATAV jobs can then query a replica database to get data

The platform architecture is depicted in Fig. 4. In order to run ATAV jobs, users log in to the head node, which automatically allocates resources and submits jobs to the cluster. A standard setup with a 6 node Sun Grid Engine (SGE) cluster (2 × 10 Cores, 128 GB RAM) allows at least 100 jobs to be run simultaneously. Each job queries a replica database with minimum database connections thus optimizing speed and workload. Using a local customized bioinformatics pipeline, it is possible to continue loading new samples into the master database which will automatically replicate to all replica databases.

### Application

The ATAV command line tool is the programmatic user interface to ATAVDB. Written in Java, ATAV consists of three modules. (1) The command line parser and query engine translate user defined parameters and the input sample list (in PLINK’s PED format [20]) into an efficient SQL query for interrogating the relational database, (2) A runtime variant object creator parses SQL output into a collection of variant objects. Each variant object includes variant information (genomic coordinates, annotation), variant calls in the sample list, sample genotype calls at coordinates without a called variant and external annotation data. (3) A statistical analysis module iterates over the variant objection collection to perform downstream analyses. ATAV currently supports tests for diagnostic analyses such as identifying putative de novo and inherited genotypes of interest in trios, and a framework for performing region-based rare-variant collapsing analyses that identify genes or other genomic units that carry an excess of qualifying variants among cases in comparison to the background variation observed in internal controls in ATAVDB. Furthermore, ATAV provides commands to output other popular formats such as PLINK’s PED/MAP files or multi-sample VCF files that can be used as input for many tools outside the ATAV framework. The modularized ATAV framework enables the continuous development of new functions that operate on sequencing/variant datasets. Critical to data integrity, all ATAV analyses include an auditable log of the software and database versions, the filter parameters used, the input sample lists used in the specific run and all runtime logs that ensure full reproducibility.

The analysts and researchers at the IGM, have run about 33,000 ATAV jobs within the last year. From a runtime perspective, 22,000 jobs completed in minutes, 8000 jobs completed in hours, and the remaining 3000 jobs completed within two days.

**Fig. 5:**
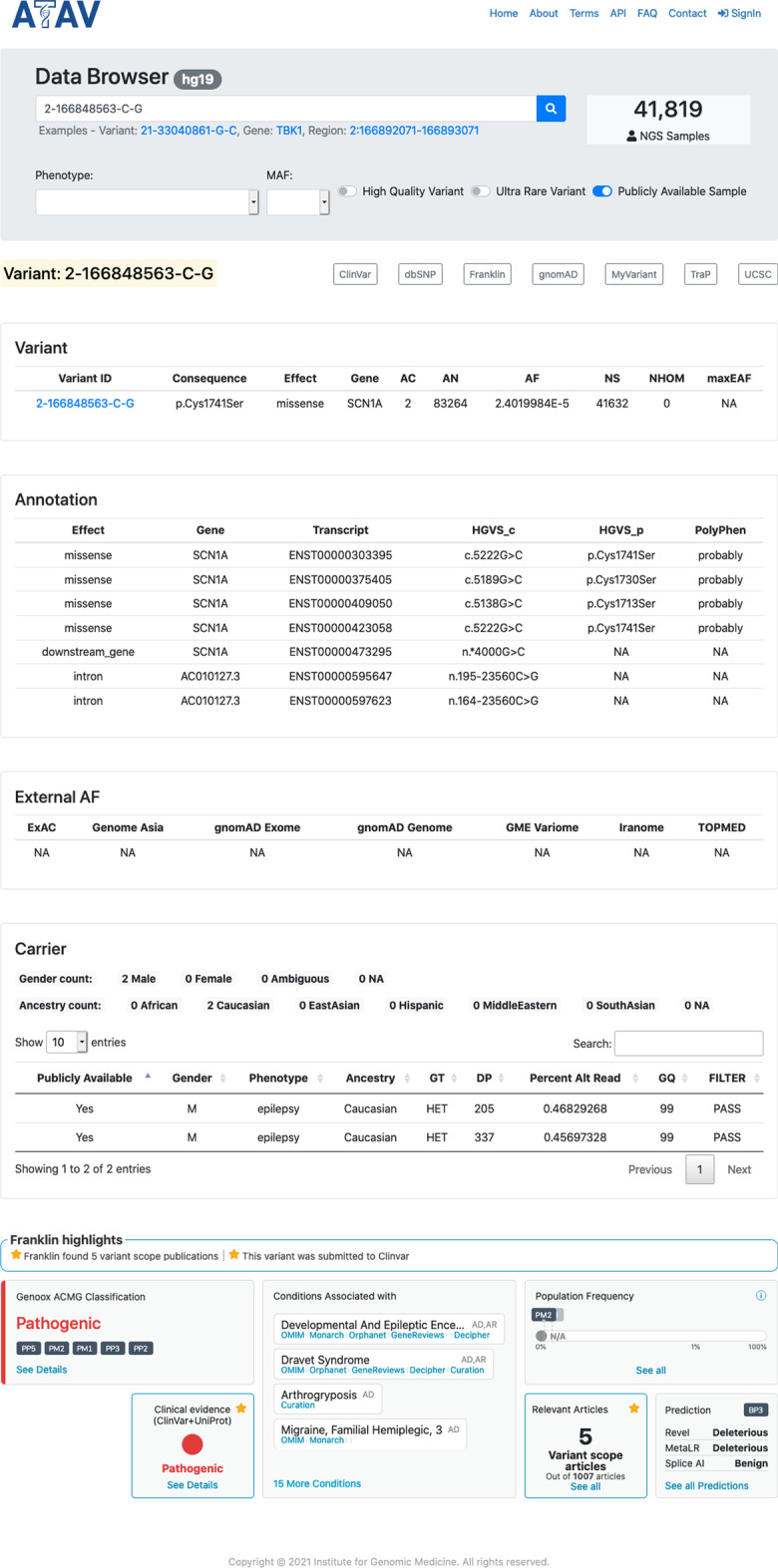
Example of the variant view of the ATAV data browser. The upper portion contains data from ATAVDB and links to public data resources while the lower portion contains data provided by the Genoox Franklin API for clinical variant interpretation

To allow access to variant-level data directly from the full dataset (for authorized users) or the publicly available dataset (for anonymous users) in ATAVDB, we provide a web-based user interface, the ATAV data browser (http://atavdb.org/). It supports the search of variants by gene, region, and variant ID. The gene or region view displays a list of variants with allele count, allele frequency, number of samples, effect, gene etc. The variant view (see Fig. 5) displays a set of annotations (effect, gene, transcript, PolyPhen [21]) and details about variant carriers (gender, predicted ancestry, phenotype, and quality metrics). It includes links to other public data resources such as Ensembl, gnomAD [3], ClinVar [16, 17], and others, and directly integrates additional annotations via APIs such as. the Genoox Franklin API for clinical variant interpretation (see Fig. 5). The data browser has several advanced filters that restrict results to rare or ultra-rare variants (using a maximum allele frequency threshold), high quality variants, or variants from samples with a specific phenotype. This variant information can also be easily queried programmatically through a REST API. The public view currently contains more than 40,000 samples representing a mix of cases and healthy controls of diverse ancestries. Users can look up potential disease-causing variants and check whether the phenotype of variant carriers in ATAVDB matches their phenotype of interest. In contrast to many other platforms, the data browser is able to show data of newly added samples in real-time and is therefore evolving rapidly as more and more samples are sequenced. While the current version only supports hg19, future updates will also include a version for hg38 once enough data has been generated.

### Collapsing analysis

ATAV provides functions for all recommended steps of the rare-variant collapsing workflow recently summarized in Povysil et al. [22]. For the sample pruning steps, ATAV creates the necessary input files by querying data from ATAVDB and automatically calls existing standard tools such as KING [23] or FlashPCA [24]. Because the coverage information for every sample and site is already efficiently stored in ATAVDB, ATAV can efficiently compare coverage between cases and controls and provides two different tests to perform coverage harmonization: sites can be removed if cases and controls show differing proportions of individuals with enough coverage [25]; or if a binomial test shows that the case/control status and coverage are not independent [26]. The outputs of the sample pruning and coverage harmonization steps can be used as inputs for dominant or recessive collapsing models. Within the collapsing model call, ATAV selects qualifying variants (QVs) that pass filters based on variant quality (Phred quality (QUAL), genotype Phred quality (GQ), quality by depth (QD), mapping quality (MQ), and variant quality score log-odds (VQSLOD)), variant annotation (effect, pathogenicity prediction scores, intolerance scores), as well as internal and external minor allele frequencies (MAFs). All QVs are used for building the collapsing matrix, a gene-by-individual indicator matrix with a value of 0 if there is no qualifying variant found in that gene in that individual, and a value of 1 if there is at least one. This collapsing matrix is used for examining associations between genes with QVs and the phenotype of interest by using a Fisher’s exact test or Firth-based logistic regression. Finally, quantile–quantile (QQ) plots are created and the genomic inflation factor lambda is estimated using a permutation-based expected distribution of *p*-values [25]. A standard collapsing analysis usually consists of several different models that all capture specific types of QVs. While quality control (QC) filters are used for all models, other filters, such as the predicted variant effects or population allele frequencies, depend on the specific model in use. In order to speed up computation, ATAV provides the option of running a general collapsing model first, using the QC filters that are shared by all models and relaxed allele frequency thresholds. The output of this initial model can be used as input for a “collapsing-lite” function that makes it possible to run the individual collapsing models within minutes because additional filters can simply be applied to the previous output and the variant database does not have to be queried again. Example commands detailing the full workflow can be found on GitHub (https://github.com/nickzren/atav/wiki/Collapsing-Workflow).

### Diagnostic analysis

ATAV also supports a diagnostic workflow, which highlights all variants in an individual that are candidates to satisfy criteria for “Pathogenic” or “Likely Pathogenic” according to the American College of Medical Genetics and Genomics (ACMG). These candidate variants can then undergo further examination by genetic counselors and a laboratory director. All annotations and filters mentioned previously, such as QC filters or internal or external MAFs, are also important for diagnostic analyses—especially for singletons where we cannot use additional family information. In addition, ATAV provides special functions for trios and families to reduce the number of potential disease-causing variants in the final output. ATAV leverages information about family structure and affectedness status that is provided by the sample file (PLINK-style PED file). Multiple families can be analyzed at once and related controls are automatically removed when calculating control frequencies. Furthermore, the affectedness status is used to decide whether to look for inherited or de novo variants. In the standard trio case of one affected offspring and two unaffected parents, ATAV uses a series of functions to extract all novel genotypes: de novo variants, newly compound-heterozygous, and newly homozygous variants. For distinguishing compound-heterozygosity from variants that are in-phase, ATAV checks that both parents carry one of the qualifying variants. ATAV not only considers the genotype of the individuals, but also their coverage. If the coverage at a variant site is below a minimum threshold of 10 for any of the individuals the variant is still included in the output, but flagged as possibly de novo, possibly newly compound-heterozygous or possibly newly homozygous. Furthermore, ATAV identifies putative parental-mosaic variant transmissions. For each parent–child pair, it extracts all variants that were transmitted from parent to child where the variant in the parent has a low proportion of alternate alleles indicating mosaicism.

ATAV also combines information from multiple variant and disease databases (e.g. ClinVar [16, 17], HGMD [19], OMIM, ClinGen [18]) into an external annotation dataset called KnownVar. The data is stored in ATAVDB and regularly updated. KnownVar annotations are not only included if the "exact" variant has been reported before, but also if a different variant at the same site has been linked to disease. Typical annotations include the associated disease, ClinVar clinical significance, HGMD Class and Pubmed IDs of relevant papers. Variants are also annotated with information extracted from HGMD and ClinVar for any disease-associated variants in close proximity. On a gene level, annotations include the total number of likely pathogenic or pathogenic variants of each category (copy number variation, small insertion/deletion, splice, nonsense, missense) in ClinVar, disease associations and inheritance from OMIM, and dosage sensitivity from ClinGen. All the information provided by KnownVar can be used as additional information in the diagnostic setting to evaluate whether a variant can be considered as diagnostic for a specific patient. Example commands for running diagnostic analyses with ATAV can be found on GitHub (https://github.com/nickzren/atav/wiki/Diagnostic-Workflow).

## Results

The collapsing framework of ATAV has enabled the confirmation of known and the discovery of novel genes in a wide range of diseases such as epilepsies [27, 28], sudden unexplained death in epilepsy [29], congenital kidney malformations [30], chronic kidney disease [31], amyotrophic lateral sclerosis [32, 33], Alzheimer’s disease [26], retinal dystrophy [34], idiopathic pulmonary fibrosis [25], and heart failure [35]. Cirulli et al. 2015 [32] used ATAV’s rare-variant collapsing framework described above to look for genes with an excess of rare, presumably deleterious variants in patients with amyotrophic lateral sclerosis compared to controls. Among the genes that reached study-wide significance were known ALS genes, such as *SOD1,* but also a novel one called *TBK1*. In a recent study [35], ATAV was used to detect a significant enrichment of rare protein-truncating variants in the *TTN* gene in patients with heart failure of mostly ischemic etiology compared with controls.

Furthermore the diagnostic framework has helped to identify both diagnostic genotypes in known genes and candidate genotypes in novel genes in a wide range of diseases including stillbirth [36], rare undiagnosed genetic disorders [37, 38], epilepsies [39–41], alternating hemiplegia of childhood [42], and chronic kidney disease [43]. Zhu et al. [37] used ATAV’s trio diagnostic pipeline to analyze 119 patients with undiagnosed genetic diseases. By restricting the analysis to de novo variants, newly compound-heterozygous or newly homozygous variants and integrating data from ClinVar, HGMD, and OMIM, the authors were able to obtain a genetic diagnosis for 29 (24%) of patients. Furthermore, they identified an enrichment of damaging de novo mutations in intolerant genes highlighting a possible way of identifying novel disease genes and expanding phenotypes for known disease genes. A recent study on causal genetic variants in stillbirth [36] used ATAV’s non-trio workflow to prioritized variants thought to be enriched for pathogenicity by focusing on variants in Mendelian disease genes from OMIM that are rare in the general population. The authors identified a diagnosis in 15 of 246 cases of stillbirth (6.1%) involving both genes that had been previously implicated in stillbirth and genes that are potential candidates for phenotypic expansion.

**Fig. 6:**
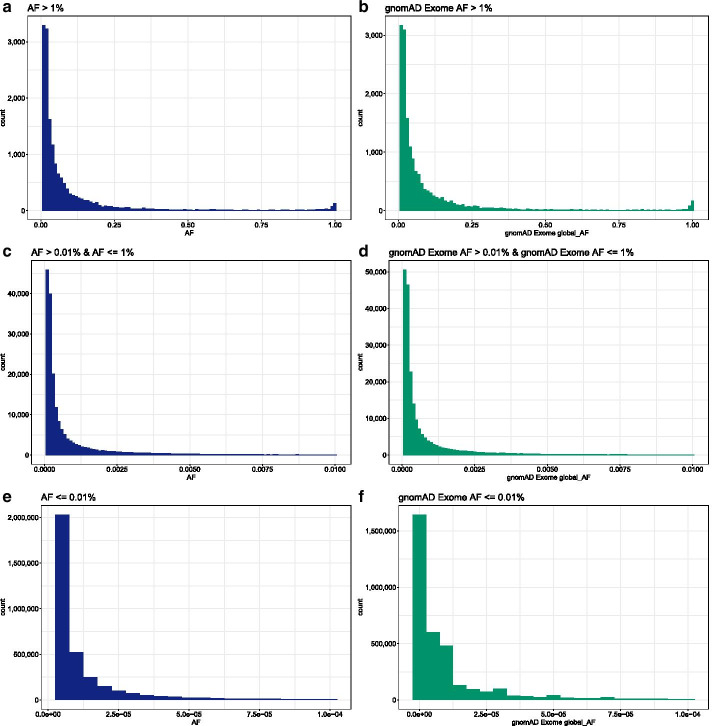
Allele frequency distributions for bi-allelic coding variants. **a**, **c**, and **e** show different AF ranges from common to extremely rare based on internal frequencies whereas the other panels show the same frequency ranges based on gnomAD v2.1 exome data. The vast majority of variants are represent singletons (left-most bar in **e**) and variants absent from gnomAD (left-most bar in **f**)

Examining the distribution of allele frequencies (AFs) can help characterize the composition of variants of the more than 100 k samples in our version of ATAVDB. Figure 6 shows AF distributions for bi-allelic coding variants, restricted to coding or splice regions that are well covered in the majority of our samples and using data of roughly 101,000 unrelated individuals. We also applied basic quality control filters including the removal of variants marked as potential artifacts by any of gnomAD’s filters. As expected, the vast majority of variants are singletons in our dataset, reflecting a frequency of 4.95 × 10^−6^ (left-most bar in panel e) and absent in the gnomAD v2.1 exome data (left-most bar in panel f).

## Conclusions

We present ATAV as an analysis platform for large-scale whole-exome and whole-genome sequencing projects. In order to encourage the widespread adoption of ATAV, we provide detailed guidelines on GitHub for how to set up the whole framework, including the database, command line tool, and data browser. This gives any user the ability to both create a local version of ATAVDB into which they can load their data and use all functionality of the command line tool and data browser in order to run analyses and queries provided by ATAV. The advantages of the ATAV framework are that (1) it allows continuous real-time analyses of all samples loaded into the database without the need for computationally demanding joint calling preceding each analysis and (2) it allows convenient logging of specific analyses performed. The newly added ATAV data browser provides easy access even to users with little computational experience by providing an intuitive web interface to query variant-level data directly from the database.

Our experience with this platform on a database carrying more than 100,000 samples indicates that a relational database can be optimized in a way that makes it possible to analyze large-scale genomic datasets. Our current data processing and storage framework is robust and flexible when combining data from multiple projects and analyzing exomes and genomes together. ATAV supports diagnostic analyses for trios and singletons, as well as rare-variant collapsing analyses for finding disease associations in complex diseases. Further optimizations are possible such as database sharding, a horizontal partition of data in a database or search engine. Other potential solutions include storing the data in Hadoop Distributed File System (HDFS) and utilizing Apache Spark to do distributed cluster computing. This would allow the processing of large amounts of variant data in parallel at once, speeding up computations and enabling an even further increase in sample sizes.

The goal of ATAV is to standardize and optimize storage and data processing for large scale sequencing data across multiple studies and to provide an easy to use interface for users with little computational experience while ensuring full reproducibility.

All code for building ATAV is publicly available, providing a convenient way for other groups to build up their own analysis platform, database, and user interface. Additionally, since we provide general access to part of our database via the ATAV browser, users can also query one of the largest variant databases available for patients sequenced at a tertiary care center. Currently, public users have access to summary-level data for more than 40,000 samples, but since the data of newly sequenced samples are added in real-time, this number grows steadily, increasing the value of the database even further as more and more samples are sequenced.

## Availability and requirements

Project name: ATAV

Project home page: https://github.com/nickzren/atav

Operating system(s): Platform independent

Programming languages: Java, Python, R, HTML and Javascript

Other requirements: Java 1.8 or higher, Percona Server 5.6 or higher

License: MIT License

Any restrictions to use by non-academics: No restrictions

## Web links and URLs

ATAV, https://github.com/nickzren/atav

ATAV data browser, http://www.atavdb.org/

ClinEff, http://www.dnaminer.com/clineff.html

ClinGen, https://clinicalgenome.org/

ClinVar, https://www.ncbi.nlm.nih.gov/clinvar/

Ensembl GRCh37, https://grch37.ensembl.org/

ExAC, http://exac.broadinstitute.org/

dbSNP, https://www.ncbi.nlm.nih.gov/snp/

Franklin, https://franklin.genoox.com/

Iranome, http://www.iranome.com/

MyVariant, http://myvariant.info/

Genome Asia, https://browser.genomeasia100k.org/

GME Variome, http://igm.ucsd.edu/gme/

gnomAD, https://gnomad.broadinstitute.org/

HGMD, http://www.hgmd.cf.ac.uk/ac/index.php

OMIM, https://www.omim.org/

TOPMed hg19, https://bravo.sph.umich.edu/freeze3a/hg19/

TraP, http://trap-score.org/

RVIS, http://genic-intolerance.org/

UCSC Genome Browser, https://genome.ucsc.edu/index.html

## Data Availability

The ATAV data browser is hosted at http://atavdb.org/. All code is freely available on GitHub at https://github.com/nickzren/atav.

## Abbreviations

VCF: Variant call format
BAM: Binary alignment map
gnomAD: The Genome aggregation database
RVIS: Residual variation intolerance score
ATAVDB: ATAV database
IGM: Institute for genomic medicine
ClinEff: Clinical Variant Annotations Software
ExAC: The Exome Aggregation Consortium
GERP: Genomic evolutionary rate profiling
TraP: The transcript-inferred pathogenicity
LIMBR: The localized intolerance model using Bayesian regression
MTR: The missense tolerance ratio
HGMD: The human gene mutation database
OMIM: Online Mendelian inheritance in man
SQL: Structured query language
PolyPhen: Polymorphism phenotyping
QC: Quality control
QVs: Qualifying variants
QUAL: Quality score
GQ: Genotype quality score
QD: Quality by depth score
MQ: Mapping quality score
VQSLOD: Variant quality score log-odds
MAF: Minor allele frequencies
HDFS: Hadoop distributed file system

## References

[1] Hout CV Van, Tachmazidou I, Backman JD, Hoffman JX, Ye B, Pandey AK, et al. Whole exome sequencing and characterization of coding variation in 49,960 individuals in the UK Biobank. bioRxiv. 2019; 572347. 10.1101/572347.

[2] Taliun D, Harris DN, Kessler MD, Carlson J, Szpiech ZA, Torres R, et al. Sequencing of 53,831 diverse genomes from the NHLBI TOPMed Program. bioRxiv. 2019; 563866. 10.1101/563866. 10.1038/s41586-021-03205-y PMC7875770 33568819

[3] Karczewski KJ, Francioli LC, Tiao G, Cummings BB, Alföldi J, Wang Q (2020). The mutational constraint spectrum quantified from variation in 141,456 humans. Nature.

[4] Petrovski S, Gussow AB, Wang Q, Halvorsen M, Han Y, Weir WH (2015). The intolerance of regulatory sequence to genetic variation predicts gene dosage sensitivity. PLoS Genet.

[5] Guðbjartsson H, Georgsson GF, Guðjónsson SA, Valdimarsson RÞ, Sigurðsson JH, Stefánsson SK (2016). GORpipe: a query tool for working with sequence data based on a Genomic Ordered Relational (GOR) architecture. Bioinformatics.

[6] Cingolani P, Platts A, Wang LL, Coon M, Nguyen T, Wang L (2012). A program for annotating and predicting the effects of single nucleotide polymorphisms. SnpEff Fly (Austin).

[7] Lek M, Karczewski KJ, Minikel EV, Samocha KE, Banks E, Fennell T (2016). Analysis of protein-coding genetic variation in 60,706 humans. Nature.

[8] Dewey FE, Murray MF, Overton JD, Habegger L, Leader JB, Fetterolf SN (2016). Distribution and clinical impact of functional variants in 50,726 whole-exome sequences from the DiscovEHR study. Science.

[9] Gelfman S, Wang Q, McSweeney KM, Ren Z, La Carpia F, Halvorsen M (2017). Annotating pathogenic non-coding variants in genic regions. Nat Commun.

[10] Hayeck TJ, Stong N, Wolock CJ, Copeland B, Kamalakaran S, Goldstein DB (2019). Improved pathogenic variant localization via a hierarchical model of sub-regional intolerance. Am J Hum Genet.

[11] Traynelis J, Silk M, Wang Q, Berkovic SF, Liu L, Ascher DB (2017). Optimizing genomic medicine in epilepsy through a gene-customized approach to missense variant interpretation. Genome Res.

[12] Gussow AB, Petrovski S, Wang Q, Allen AS, Goldstein DB (2016). The intolerance to functional genetic variation of protein domains predicts the localization of pathogenic mutations within genes. Genome Biol.

[13] Ioannidis NM, Rothstein JH, Pejaver V, Middha S, McDonnell SK, Baheti S (2016). REVEL: an ensemble method for predicting the pathogenicity of rare missense variants. Am J Hum Genet.

[14] Sundaram L, Gao H, Padigepati SR, McRae JF, Li Y, Kosmicki JA (2018). Predicting the clinical impact of human mutation with deep neural networks. Nat Genet.

[15] Havrilla JM, Pedersen BS, Layer RM, Quinlan AR (2019). A map of constrained coding regions in the human genome. Nat Genet.

[16] Landrum MJ, Lee JM, Riley GR, Jang W, Rubinstein WS, Church DM (2014). ClinVar: public archive of relationships among sequence variation and human phenotype. Nucleic Acids Res.

[17] Landrum MJ, Lee JM, Benson M, Brown GR, Chao C, Chitipiralla S (2018). ClinVar: improving access to variant interpretations and supporting evidence. Nucleic Acids Res.

[18] Rehm HL, Berg JS, Brooks LD, Bustamante CD, Evans JP, Landrum MJ (2015). ClinGen: the clinical genome resource. N Engl J Med.

[19] Stenson PD, Ball EV, Mort M, Phillips AD, Shiel JA, Thomas NST (2003). Human gene mutation database (HGMD^®^): 2003 update. Hum Mutat.

[20] Purcell S, Neale B, Todd-Brown K, Thomas L, Ferreira MAR, Bender D (2007). PLINK: a tool set for whole-genome association and population-based linkage analyses. Am J Hum Genet.

[21] Adzhubei IA, Schmidt S, Peshkin L, Ramensky VE, Gerasimova A, Bork P (2010). A method and server for predicting damaging missense mutations. Nat Methods.

[22] Povysil G, Petrovski S, Hostyk J, Aggarwal V, Allen AS, Goldstein DB (2019). Rare-variant collapsing analyses for complex traits: guidelines and applications. Nat Rev Genet.

[23] Manichaikul A, Mychaleckyj JC, Rich SS, Daly K, Sale M, Chen W-M (2010). Robust relationship inference in genome-wide association studies. Bioinformatics.

[24] Abraham G, Qiu Y, Inouye M (2017). FlashPCA2: principal component analysis of Biobank-scale genotype datasets. Bioinformatics.

[25] Petrovski S, Todd JL, Durheim MT, Wang Q, Chien JW, Kelly FL (2017). An exome sequencing study to assess the role of rare genetic variation in pulmonary fibrosis. Am J Respir Crit Care Med.

[26] Raghavan NS, Brickman AM, Andrews H, Manly JJ, Schupf N, Lantigua R (2018). Whole-exome sequencing in 20,197 persons for rare variants in Alzheimer’s disease. Ann Clin Transl Neurol.

[27] Allen AS, Bellows ST, Berkovic SF, Bridgers J, Burgess R, Cavalleri G (2017). Ultra-rare genetic variation in common epilepsies: a case-control sequencing study. Lancet Neurol.

[28] Zhu X, Padmanabhan R, Copeland B, Bridgers J, Ren Z, Kamalakaran S (2017). A case-control collapsing analysis identifies epilepsy genes implicated in trio sequencing studies focused on de novo mutations. PLoS Genet.

[29] Bagnall RD, Crompton DE, Petrovski S, Lam L, Cutmore C, Garry SI (2016). Exome-based analysis of cardiac arrhythmia, respiratory control, and epilepsy genes in sudden unexpected death in epilepsy. Ann Neurol.

[30] Sanna-Cherchi S, Khan K, Westland R, Krithivasan P, Fievet L, Rasouly HM (2017). Exome-wide association study identifies GREB1L mutations in congenital kidney malformations. Am J Hum Genet.

[31] Cameron-Christie S, Wolock CJ, Groopman E, Petrovski S, Kamalakaran S, Povysil G (2019). Exome-based rare-variant analyses in CKD. J Am Soc Nephrol.

[32] Cirulli ET, Lasseigne BN, Petrovski S, Sapp PC, Dion PA, Leblond CS (2015). Exome sequencing in amyotrophic lateral sclerosis identifies risk genes and pathways. Science.

[33] Gelfman S, Dugger S, de Araujo Martins-Moreno C, Ren Z, Wolock CJ, Shneider NA (2019). A new approach for rare variation collapsing on functional protein domains implicates specific genic regions in ALS. Genome Res.

[34] Wolock CJ, Stong N, Ma CJ, Nagasaki T, Lee W, Tsang SH (2019). A case–control collapsing analysis identifies retinal dystrophy genes associated with ophthalmic disease in patients with no pathogenic ABCA4 variants. Genet Med.

[35] Povysil G, Chazara O, Carss KJ, Deevi SVV, Wang Q, Armisen J (2020). Assessing the role of rare genetic variation in patients with heart failure. JAMA Cardiol.

[36] Stanley KE, Giordano J, Thorsten V, Buchovecky C, Thomas A, Ganapathi M (2020). Causal Genetic Variants in Stillbirth. N Engl J Med.

[37] Zhu X, Petrovski S, Xie P, Ruzzo EK, Lu Y-F, McSweeney KM (2015). Whole-exome sequencing in undiagnosed genetic diseases: interpreting 119 trios. Genet Med.

[38] Petrovski S, Shashi V, Petrou S, Schoch K, McSweeney KM, Dhindsa RS (2015). Exome sequencing results in successful riboflavin treatment of a rapidly progressive neurological condition. Mol Case Stud.

[39] Allen AS, Berkovic SF, Cossette P, Delanty N, Dlugos D (2013). De novo mutations in epileptic encephalopathies. Nature.

[40] Myers CT, Stong N, Mountier EI, Helbig KL, Freytag S, Sullivan JE (2017). De Novo mutations in PPP3CA cause severe neurodevelopmental disease with seizures. Am J Hum Genet.

[41] Petrovski S, Küry S, Myers CT, Anyane-Yeboa K, Cogné B, Bialer M (2016). Germline de Novo mutations in GNB1 cause severe neurodevelopmental disability, hypotonia, and seizures. Am J Hum Genet.

[42] Heinzen EL, Swoboda KJ, Hitomi Y, Gurrieri F, De Vries B, Tiziano FD (2012). De novo mutations in ATP1A3 cause alternating hemiplegia of childhood. Nat Genet.

[43] Groopman EE, Marasa M, Cameron-Christie S, Petrovski S, Aggarwal VS, Milo-Rasouly H (2019). Diagnostic utility of exome sequencing for kidney disease. N Engl J Med.

